# Effect of Oral Vitamin D3 on Dental Caries: An In-Vivo and In-Vitro Study

**DOI:** 10.7759/cureus.25360

**Published:** 2022-05-26

**Authors:** Sawsan Hameed Al-Jubori, Maha A AL-Murad, Faehaa Azher Al-Mashhadane

**Affiliations:** 1 Department of Conservative Dentistry, University of Mosul, Mosul, IRQ; 2 Department of Dental Basic Sciences, University of Mosul, Mosul, IRQ

**Keywords:** vitamin d, tooth remineralization, tooth demineralization, saliva, dental caries

## Abstract

Aim

Vitamin D3 plays an important role in affecting the overall remineralization process of the dentition. The use of supplements help to keep the levels at optimum and thus reduce the chances of treating very early lesion of caries. Hence the aim was to investigate the indirect effects of oral vitamin D3 on microhardness and elemental weight percentage of Calcium (Ca) and Phosphorous (P) in enamel surface with an artificially initiated carious lesion.

Methods

The 120 extracted premolars were randomly divided into five groups according to salivary immersion. Each group had a total of twenty-four participants, with the following characteristics: control +ve: sound enamel; control -ve: only subjected to pH cycle; A: pH cycle and immersion in control saliva; B: pH cycle and saliva collected after three weeks; and C: pH cycle and saliva collected after six weeks. The unstimulated saliva was collected from (40) adult volunteers receiving vitamin D3 1000IU gel capsules daily for six weeks. Before each vitamin D3 intake, 10 mL of unstimulated control saliva was collected from each participant. Then other 10 ml. were collected after three and six weeks of vitamin D receiving periods. Saliva immersion time (12 hours). Enamel surface was assessed by Vicker’s Microhardness machine and (X-ray fluorescence - XRF) spectrometer.

Results

For all specimens, there was a significant decrease in both (Ca and P weight %) after demineralization and then they significantly increased after receiving vitamin D3. The microhardness and elemental analysis provide confirmed results that were represented as a statistically significant difference at (P≤ 0.05) between groups that received vitamin D3 and those without vitamin D3 dosage.

Conclusions

Oral vitamin D3 has a significant potential in motivating remineralization of early lesions on the enamel surfaces representing improved surface microhardness and minerals content (Ca and P weight %) of demineralized tooth surfaces.

## Introduction

Dental caries are usually initiated as a white spot lesion on the enamel as an early sign of demineralization [1]. The effective and non-invasive treatment could enhance the process of remineralization and help in the maintenance of healthy dentition [2]. Currently, the selection of a good treatment method to remineralize hard tissue and reduce the progress or regression of any lesion is the key to preventing invasive treatment procedures [3]. Microhardness measurement is a simple and dependable method to determine the mechanical properties of enamel following changes in mineral content [4]. Frequently, in non-destructive chemical assessments of minerals, sediments, and fluids an X-ray fluorescence (XRF) spectrometer is utilized. It is the widely used best analytical technique to perform elemental analysis in different types of samples with XRF microscopy is feasible for more accurate estimations of tooth demineralization [5].

Parthasarathy et al. [6] analyzed the relationship between dental caries and vitamin D3 and indicated that the receptors for vitamin D3 are present in cells of the immune system which binds to vitamin D3 and increases the formation of antimicrobial protein which prevent dental caries. The ameloblast and odontoblast cells that form enamel and dentin, respectively, have vitamin D3 receptors which support lowering the risk of tooth decay [7].

Vitamin D3 plays a pivotal role in many biological functions like calcium (Ca) and phosphorous (P) metabolism and hard tissue mineralization and tooth formation [8]. Thus, the current research was carried out to assess the indirect effect of vitamin D3 (oral administration) on remineralization of early initiated enamel caries lesion in human permanent teeth through evaluation of enamel surface microhardness, and elemental weight percentage of Ca and P in teeth surface using XRF analysis. The null hypothesis was that there was no significant difference in teeth enamel surface microhardness and elemental weight percentage of Ca and P between groups with and without vitamin D3 (oral administration) in different time intervals.

## Materials and methods

This study's experimental work took place in the College of Dentistry, University of Mosul, Iraq, from November 2019 to June 2020. This randomized clinical trial selected 40 participants (20 females, 20 males) of the age range of (20-40 years) with no history of vitamin D3 allergy, any systemic diseases, non-pregnant or lactating females, no drug or supplements for the last (three) months, non-smoker, and non-alcoholic, and they agreed to contribute to this study by signing a consent form.

Informed consent was obtained by all participants in this study. The study was a randomized clinical trial, approved by the Scientific Academic and Research Ethical Committee of the Department of Dental Basic Sciences, College of Dentistry, the University of Mosul (issued approval 3rd session 11/17/2019).

All volunteers received Vitamin D3 (1000 IU) fast-acting liquid soft gel capsules** **daily for six weeks.

Saliva collection and storage

Unstimulated saliva samples were collected as follows: The participants were seated on a standardized condition and their scaling and polishing were done to reach the baseline. On the day next to scaling and polishing, 10 ml. of unstimulated saliva was collected before receiving Vitamin D3 dosage. Before saliva collection, the patients were instructed to rinse their oral cavity with water. The collection saliva time was about 10-20 minutes and was done 2 hr after having breakfast. Other saliva samples (10 ml) were obtained once more on the twenty-first day after the treatment started. The third stage of saliva samples (10 ml) were also taken on the forty-second day after the treatment starts for each participant. The saliva samples were frozen at or below -20ºC until the time of use as all the samples were to be collected and sent at once.

For the in vitro study, the saliva samples were brought to room temperature, then clear saliva samples were obtained by centrifugation of all samples for the in vitro part of the study.

The 120 extracted premolars from orthodontic patients in the College of Dentistry, University of Mosul were used and examined with radiograph and stereomicroscope for any caries, restorations, hypoplastic lesions, cracks, and/or white spot lesions. With non-fluoridated pumice and de-ionized water, each tooth was cleaned and polished with a slow-speed handpiece and rubber cup. Using a slow-speed diamond disc (Drendel-Zweiling, Germany), all crowns were cut about 1 mm below the cementoenamel junction. Then were preserved in a thymol solution (0.1%) till the experimental procedure.

Preparation of demineralizing and remineralizing solutions

Demineralizing and remineralizing solutions, and pH adjustment were identified by Featherstone et al. [9]. Then modified by many researchers for more safe and simplified procedures used for induction of initial caries-like lesions in the enamel surface. The solution of demineralization contains “0.075 mM/L acetic acid, 1 mM/L calcium chloride, and 2 mM/L potassium phosphate” and the pH will be adjusted to 4.3. Whereas the solution of remineralization, which contains “150 mM/L potassium chloride, 1.5 mM/L calcium nitrate, and 0.9 mM/L potassium phosphate” and the pH will be adjusted to 7 [10].

pH cycling procedure

To simulate acidic challenge in a daily manner inside the environment of the mouth, crown samples were soaked in a demineralization solution for six hours, then were rinsed with distilled water for (1 minute) and then immersed in the prepared remineralizing agent for 17 hours. These steps were repeated once per day for 10 days [10]. The demineralizing and remineralizing solutions were refreshed daily. A total of 10 cycles were conducted.

Division of groups

The crowns were randomly divided into five groups, each group consisted of 12 samples for microhardness and 12 samples for the XRF analyses.

Control +ve: The crowns with no treatment (sound enamel) stored in distilled water; control -ve: the crowns that were subjected to a pH cycle and then stored in distilled water.

The remaining crowns were subjected to a pH cycle, were washed with distilled water for 1 minute, then immersed in one of the saliva groups for 12 hours.

Group A: The control baseline saliva that was collected from patients before vitamin D3 oral administration; Group B: the saliva that was collected on the twenty-first day of vitamin D3 administration; Group C: the saliva that was collected on the forty-second day.

Finally, surface microhardness and elemental analysis (XRF) were performed.

Microhardness analysis

The 60 crowns were embedded in self-cured acrylic resin blocks with labial surfaces exposed, facing upward, in a direction parallel to the horizontal plane. Then by using 400, 800, 1000, and 1200 grit abrasive paper, the buccal surface of each sample was flattened and was polished sequentially to produce a more consistent reproducible flat enamel surface, then was subjected to pH cycling according to grouping [11].

Surface microhardness was tested with the microhardness testing machine (Vickers Wolpert, Germany) for samples. The indentations were created with Vibromechanical texturing (VMT) at the rate of 100 g load for 10 seconds. Three indentations were used to determine the sample's average microhardness, then get average microhardness mean value for each group was calculated.

The Vickers hardness number (VHN) was calculated using the following formula: [12]

HV = 1.854 P/d2 

Where HV = Vickers hardness in Kg/mm2; P = the load applied in Kg; d = the average length of the diagonals in mm; 1.854 = a constant number.

Quantitative elemental analysis (weight %) by XRF

Both Ca and P content were quantified in the 60 crowns using XRF (Pw 1410, Phillips, The Netherlands), a computer-controlled software programme with an accelerating voltage of 15 Kv, X-ray intensities in counts per second were recorded, and electron beams were maintained at a constant distance (2 x10-10 amp) to record the values. Elemental levels including Ca and P were evaluated in weight percentage.

Statistical analysis

The SPSS® 25.0 software (IBM Corp, Armonk, USA) was used to conduct the statistical analysis. The normality test presented a normal (parametric) distribution. One-way ANOVA was used to compare the groups. Tukey's Honest Significant Difference (HSD) post hoc test was used for pair-wise comparisons. All analyses were performed with a significance level set at p ≤ 0.05.

## Results

Microhardness analysis

The ANOVA test showed that there is a highly significant difference in microhardness of tested groups at p ≤ 0.05 (Table 1).

**Table 1:** Groups were analyzed according to Vickers hardness number (VHN) *significant at p ≤ 0.05 F: Fischer F distribution value; Sig.: significance

	Sum of Squares	Mean Square	F	Sig.
Group A vs B vs C vs Sound enamel vs crowns with change in pH cycle	967.068	241.767	146.441	.000*
Within Groups A, B, C, Sound enamel, crowns with change in pH cycle	90.803	1.651		

The results also revealed that the Control +ve group had the statistically significantly highest hardness (72.7200 VHN), while the demineralized group Control -ve group had the lowest microhardness (63.1982 VHN) but with no significant difference compared to Group A. And there is no significant difference between Group B = 69.9225 and Group C = 70.9505 VHN. Both showed a statistically significantly higher microhardness than that of Control -ve and Group A and lower microhardness than Control +ve group. Concerning the periods of oral administration, there are no significant differences in microhardness between Group B and Group C, which represent three and six weeks, respectively (Table 2). 

**Table 2:** Comparison of groups according to Vickers hardness number (VHN) (Tukey HSD test) ^A, B, C^ Pair comparison leading to statistical significance; different letters in the same column indicate statistical significance (p <0.05). (Tukey HSD test shows ^A, B, C  ^ that values with the same letter are nonsignificant) HSD: Honest Significant Difference

Groups	N	Mean	Std. Deviation
Control +ve (sound enamel)	12	72.7200^C^	1.50403
Control -ve (Crown subjected to pH cycle)	12	63.1982^A^	1.68279
A (pH cycle+ control saliva)	12	63.2197^A^	.89177
B (pH cycle+ saliva after 3 weeks)	12	69.9225^B^	1.41786
C (pH cycle+ saliva after 6 weeks)	12	70.9505^B^	.59609

Quantitative elemental analysis (weight %) by XRF

The ANOVA test for mineral types at p ≤ 0.05 is listed in (Table 3). The Ca and P weight % (wt%) represent highly significant differences between and within tested groups.

**Table 3:** Analysis of variance (ANOVA) test for mineral types. *significant at p ≤ 0.05 df: degrees of freedom; sig.: significance; F: Fischer F distribution value

Mineral		Sum Squares	df.	Mean Square.	F	Sig.
Calcium	Between Groups	73.820	4	18.455	26805.276	.000*
	Within Groups	.038	55	.001		
	Total	73.858	59			
Phosphorus	Between Groups	12.029	4	3.007	6265.361	.000*
	Within Groups	.026	55	.000		
	Total	12.056	59			

The result represents the amount of Ca and P wt% present in the samples and the changes in the concentration of the mineral components of the different groups,

Tukey's HSD test results showed that the Control +ve group had the statistically significant highest Ca wt% (12.0300) in comparison to other groups, except Group C, while the demineralized specimens of the Control -ve group had statistically significant lowest Ca wt% (9.5200). Groups A and B represent significant differences; both showed statistically significantly higher Ca wt% than the demineralized group Control -ve group and significantly lower Ca wt% than that of the sound enamel control group.

The P wt% represents a highly significant difference among all tested groups as according to the Tukey HSD test, the Control -ve group showed a significantly lower P wt% than that of the sound enamel (Control +ve) group which represents the highest mean. There are significant differences in the elemental weight percentage of Ca and P in Group B and C that are related to administration periods (Table 4).

**Table 4:** Comparative values of Calcium and Phosphorus wt% of the different groups in XRF analysis. ^A, B, C, D, E^ Pair comparison leading to statistical significance; different letters in the same column indicate statistical significance (p < 0.05) (Tukey HSD test shows ^A, B, C, D, E^  that the values with the same letter are nonsignificant) XRF: X-ray fluorescence

Mineral	Group	N	Mean wt%	Std. Deviation
Calcium	Control +ve (sound enamel)	12	12.0300^D^	.01706
	Control -ve (Crown subjected to pH cycle)	12	9.5200^A^	.01477
	A (pH cycle+ control saliva)	12	9.7200^B^	.02256
	B (pH cycle+ saliva after 3 weeks)	12	10.1633^C^	.04376
	C (pH cycle+ saliva after 6 weeks)	12	12.0200^D^	.02256
Phosphorus	Control +ve (sound enamel)	12	1.9933^E^	.03447
	Control -ve (Crown subjected to pH cycle)	12	.7767^A^	.01969
	A (pH cycle+ control saliva)	12	0.9967^B^	.00492
	B (pH cycle+ saliva after 3 weeks)	12	1.3700^C^	.01706
	C (pH cycle+ saliva after 6 weeks)	12	1.7200^D^	.02256

## Discussion

Dental caries have often been considered with multifactorial etiology and multiple risk factors. The body's antioxidant processes include several substances that can lower oxidative stress and free radical products. Salivary antioxidants may be able to serve as the initial line of defense to control the effects of oxidative stress. The oxidative stress will be countered by a collection of salivary enzymes that includes salivary peroxidase, salivary uric acid, and other minor enzymes [13]. 

Recently, extensive literature has been produced on vitamin D3 for its important role in dental and general human body health [14]. The development of a remineralization protocol is the objective of current dentistry to improve the appearance, strength, and function of teeth by preventing caries progression [1]. The presence of calcium and phosphorus in demineralizing solutions keeps the surface enamel layer intact while minerals from the subsurface layer are lost, therefore adding these minerals with acetic acid "weak acid" for this purpose is a good idea [15].

In the present study, changes in Ca, P contents (wt%) and microhardness of teeth during the experiment were significantly decreased after exposure to the demineralization procedure while they significantly increased after immersion in saliva collected after different periods of vitamin D administration (three and six weeks); this indicates the efficacy of vitamin D3 indirectly for the remineralization of incipient enamel caries-like lesions.

So, the null hypothesis was rejected. It was established there were significant differences between enamel surface microhardness and elemental weight percentage of Ca and P after and before vitamin D3 administration.

Concerning the effect of the periods of vitamin D3 oral administration, it is not clearly distinguished on the surface microhardness but it is represented in changes in Ca and P contents. This may be because we need more time, and the microhardness test is not as sensitive as XRF analysis. The challenge in the present study is that there are no similar related studies for direct comparison of the results for agreements or disagreements, so we depend indirectly on the studies that provide support for the action and effect of vitamin D3 in maintaining dental health and minerals deposition.

Vitamin D3 plays a major role in the regulation of calcium and phosphorus absorption and in maintaining skeletal and dental tissues [16-18]. Deficiency of vitamin D3 causes the delay of teeth eruption and abnormal calcification of enamel and dentin. Since vitamin D has a major function in teeth development, any defect in tooth enamel surfaces induced by such vitamin deficiency makes the tooth more susceptible to caries [19].

Mellanby et al. [20] described that children consuming cereals fortified with vitamin D3 had significantly decreased teeth decay incidences and hypoplasia. This may be related to the level of LL37. The role of vitamin D3 in cathelicidin expression, as well as the LL37 levels, is significant (a 37-amino acid peptide generated from proteolytic cleavage of the extracellular domain of the 18-kDa hCAP18 protein from epithelial cells and neutrophils), which is the only antibacterial peptide made from cathelicidin in humans [21].** **Gyll et al. [8] stated that children with insufficient D3 vitamin levels had saliva LL37 concentrations lower than those who had sufficient serum levels at follow-up. Moreover, recent studies have given evidence for the relations between vitamin D supplementation, tooth development, and caries. Mellanby and colleagues studied the relationship of the actual levels of circulating 25-hydroxy vitamin D3 (25(OH)D3) for determining vitamin D3 status [20]. According to Ahmadi-Motamayel, patients with active dental caries showed higher oxidative stress markers in a significant manner compared to the healthy control individuals [22].

Malonaldehyde, a marker of oxidative stress as the end result of a chain reaction of lipid peroxidation, can be produced by dental caries, resulting in a reduction in antioxidant levels, causing more caries progression. So, there's a clear correlation between salivary total antioxidant capacity and tooth caries [13].

For the past two decades, the relevance of antioxidants in the etiology and progression of dental caries has been a subject of controversy. The impact of vitamins like vitamin D3 and its high antioxidant properties was the focus of early investigations on antioxidants that can play a preventive role in the development of dental caries [23-25].

In agreement with our results, recent studies said that deficiency in vitamin D3 has been linked to major changes in tooth tissues. Enamel and dentin abnormalities have been considered in children as caries risk factors. As a result, optimum vitamin D3 levels in children are required as a secondary preventive intervention for dental cavities in the permanent dentition [26]. Another study concluded that among the vitamin D3 receptor polymorphisms studied, a relation was found between the FokI polymorphism (rs10735810) and the risk of dental caries, with the protective effect of the f allele and ff genotype [27].

A limitation of the study is that no experiment was done involving the dentin. Also, this study did not include the pediatric population among which dental caries are extremely prevalent.

## Conclusions

When compared to the effect of saliva without vitamin D supplements, this study concluded that saliva collected from people who received vitamin D could provide an obvious remineralization effect on initial enamel surface lesions representing the increasing Ca and P contents (wt %) and microhardness of artificially induced dental caries. This suggests that vitamin D3 supplementation has a positive impact on caries remineralization.
